# The state of the science of interprofessional collaborative practice: A scoping review of the patient health-related outcomes based literature published between 2010 and 2018

**DOI:** 10.1371/journal.pone.0218578

**Published:** 2019-06-26

**Authors:** May Nawal Lutfiyya, Linda Feng Chang, Cynthia McGrath, Clark Dana, Martin S. Lipsky

**Affiliations:** 1 College of Dental Medicine, Roseman University of Health Sciences, South Jordan, Utah, United States of America; 2 Department of Family and Community Medicine, University of Illinois-Chicago, College of Medicine at Rockford, Rockford, Illinois, United States of America; 3 Saint Anthony College of Nursing, Rockford, Ilinois, United States of America; 4 Office of the Chancellor, Roseman University of Health Sciences, South Jordan, Utah, United States of America; University of British Columbia, CANADA

## Abstract

**Introduction:**

If interprofessional collaborative practice is to be an important component of healthcare reform, then an evidentiary base connecting interprofessional education to interprofessional practice with significantly improved health and healthcare outcomes is an unconditional necessity. This study is a scoping review of the current peer reviewed literature linking interprofessional collaborative care and interprofessional collaborative practice to clearly identified healthcare and/or patient health-related outcomes. The research question for this review was: What does the evidence from the past decade reveal about the impact of Interprofessional collaborative practice on patient-related outcomes in the US healthcare system?

**Materials and methods:**

A modified preferred reporting items for systematic reviews and meta-analyses (PRISMA) approach was followed.

**Results:**

Of an initial 375 articles retrieved 20 met review criteria. The most common professions represented in the studies reviewed were physicians, pharmacists and nurses. Primary care was the most common care delivery setting and measures related to chronic disease the most commonly measured outcomes. No study identified negative impacts of interprofessional collaborative practice. Eight outcome categories emerged from a content analysis of the findings of the reviewed studies.

**Conclusions:**

The results suggest a need for more research on the measurable impact of interprofessional collaborative practice and/or care on patient health-related outcomes to further document its benefits and to explore the models, systems and nature of collaborations that best improve population health, increase patient satisfaction, and reduce cost of care.

## Introduction

Stimulated by a multitude of factors, including a heightened commitment to reforming healthcare delivery, attention to interprofessional practice and education has grown exponentially over the past decade. [1, 2]. [2–4] In 2008 Berwick et al., published their seminal article on the triple aim, offering a roadmap for healthcare delivery reform. [5] Along with the Institute of Medicine’s (IOM) 2001 report, *Crossing the Quality Chasm*,[6] and the World Health Organization’s (WHO) 2013 report *Transforming and scaling up health professionals’ education and training*,[7] these articles galvanized a renewed interest in interprofessional practice and education. Conceptually, these three works explicitly promoted interprofessional healthcare teams as a strategy to improve health services and outcomes. The IOM and WHO reports charged health professions’ programs to incorporate interprofessional education (IPE) into their training with the conviction that these efforts would lead to enhanced communication and care coordination to advance population health, reduce healthcare costs, and improve patient health-related outcomes.

Ultimately, if interprofessional practice is to be an important *component* of healthcare reform, then an evidentiary base connecting interprofessional collaborative practice (IPCP) with significantly improved health and healthcare outcomes is an unconditional necessity. [2, 3, 8] The 2013 WHO report gave a conditional recommendation of IPE, suggesting rigorous research be included in its implementation. Continued interprofessional education endeavors can only be justified if the return on investment from an IPCP model yields a positive impact on measurable patient health-related outcomes.

A 2014 scoping review [3] noted that despite its multi-decade history, interprofessional practice and education-related research focused mainly on short-term changes such as improved knowledge, skills and attitudes of learners or on intermediate policy changes in either education or clinical settings, but not on patient health and/or healthcare related outcomes.[3] As Brandt et.al. concluded “… little of the literature reviewed focused on population health or patient health outcomes, and none on the reduction in the cost of healthcare.” [3] A 2016 editorial, [4] describing the key findings of the 2015 IOM’s report on interprofessional practice and education, concluded that whether or not interprofessional practice and education improves clinical outcomes remains uncertain since few studies explicitly map interprofessional practice and education to health related outcome measures. [4]

Since most health professions programs, including pharmacy, medicine, public health, nursing and dentistry, [9–12] mandate IPE it is understandable that most research focused primarily on educational methods and learner outcomes. However, this leaves a gap in the evidence linking interprofessional practice and/or care to patient health-related outcomes. As embedded as interprofessional education is in health professions training, for justify these efforts, research must extend to include health-related patient outcomes. Yet despite the mandate for IPE, as John Gilbert notes it remains, “a great truth awaiting scientific confirmation.” An updated WHO report also supported the need for more evidence connecting IPE training to improved health outcomes.

This study is a scoping review [13] of the current peer-reviewed literature that connects interprofessional collaborative care and IPCP to clearly identified patient health-related outcomes. Scoping reviews are a process of mapping an existing literature or evidence base to answer a question. [13] For this study we explored the question: What does the evidence over the past decade reveal about the effectiveness of interprofessional collaborative care and/or interprofessional collaborative practice in the US healthcare system on patient related outcomes? In addition, the study also sought to identify gaps in the existing literature to help guide future research.

## Materials and methods

For this review *interprofessional collaborative care* was defined as: the provision of comprehensive health services to patients by multiple caregivers from different professions (e.g., medicine, nursing, pharmacy, dentistry) who work collaboratively to deliver quality care within and across settings (e.g., hospital, primary care, dental clinics, hospice care). *Interprofessional collaborative practice* occurs when healthcare providers work with people from within their own profession, with people outside their profession and with patients and their families. When healthcare providers work collaboratively, they seek common goals and can analyze and address any problems that arise. Care is coordinated according to patients’ needs and patient outcomes are explicitly tied to how care is provided (although without exception the influences on patient outcomes are multifactorial).

The review followed a modified preferred approach for reporting items for systematic reviews and meta-analyses (PRISMA). [14] The PRISMA approach is organized by five distinct elements or steps: beginning with a clearly formulated question, using the question to develop clear inclusion criteria to identify relevant studies, an approach to appraise the studies or a subset of the studies, a summary of the evidence using an explicit methodology, and interpreting the findings of the review.

The literature search was limited to peer-reviewed articles, from the time span of 2010–2018. The choice of the year 2010 as the floor was grounded in the historical fact that the US Affordable Care Act [15] became law during that year and 2018 chosen as the ceiling year because that was the last full year that had ended when the review commenced. Unpublished/grey literature, opinion pieces/essays, letters to the editor, and review papers were excluded from this review. The literature search was further limited to papers written in English and based in the US. The rationale to focus solely on US based literature is that the US-based health care system of practice and reimbursement infrastructure are unique compared to other industrialized nations. Until the enactment of the Affordable Care Act, many low to moderate-income families didn’t have access to medical home. The U.S. healthcare team is also more specialty based and focuses more primarily on clinician/patient model and not on team-based health care or universal insurance. This review aims to assess the impact of IPCP within the US healthcare system on patient related outcomes.

PubMed and Google Scholar were systematically searched to identify potentially relevant literature. Google Scholar was included because there is a growing literature [16, 17] assessing the value of Google Scholar in relationship to other indexing databases such as PubMed. Current recommendations suggest including Google Scholar and PubMed for comprehensive health-related systematic review searches [18]. We decided to use both MeSH and broader terms in our search to capture all relevant studies. The initial search terms used for the review entailed the following:

1. Interprofessional Collaborative Care in the US Healthcare;
2. Interprofessional Collaborative Practice in the US Healthcare;
3. Patient Outcomes and Interprofessional Collaborative Care in the US; and
4. Patient Outcomes and Interprofessional Collaborative Practice in the US.
  In keeping with the PRISMA approach the following five steps were adhered to.

***Step 1*:** The initial research question for this review was:

What does the outcome-derived evidence indicate about *interprofessional collaborative care* and/or *interprofessional collaborative practice* in the US healthcare system?

As the review of the literature was underway, two additional questions were formulated to guide the analyses. These were:

1. What patient health-related outcomes were measured in the literature reviewed?
2. What were the data-driven findings derived from the reviewed literature?

***Step 2*:** The initial question directed the process for identifying the relevant work reviewed. The subsequent questions guided the analysis of the reviewed literature. The inclusion criteria the review proposed emerged directly from the question guiding the review.

**Fig 1:**
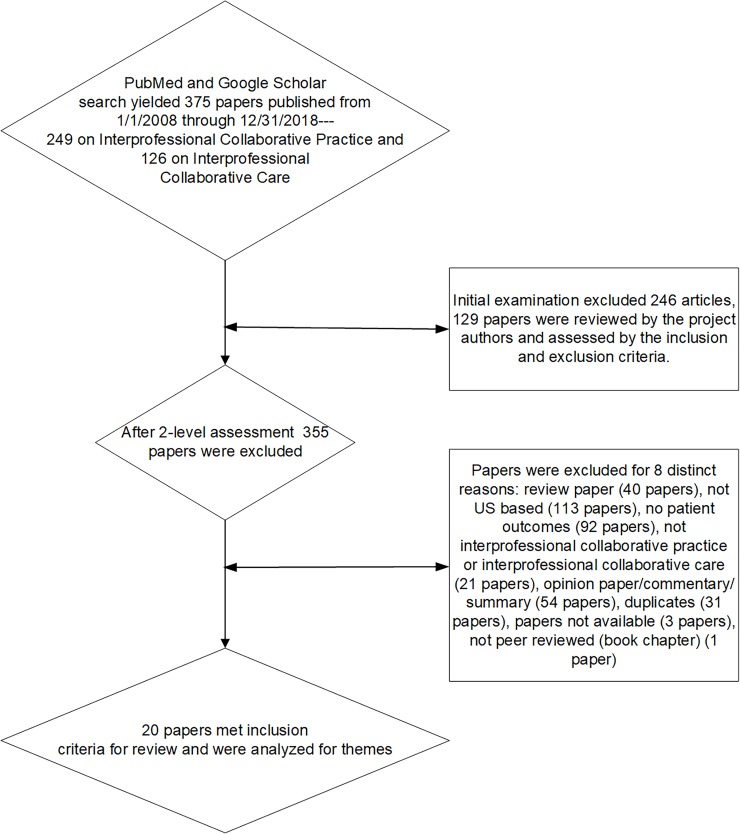
Article selection process for review.

***Step 3*:** A systematic approach to appraise the studies was used. To assess the selected papers a comprehensive table that included the full paper citation and complete abstract along with the following five components comprising a checklist was developed and used: 1) Interprofessional Collaborative Care *OR* Interprofessional Collaborative Practice (yes/no), 2) United States (yes/no), 3) Practice Setting (e.g., acute, primary, hospice), 4) Peer-Reviewed (yes/no), 5) study design specified (yes/no), and 6) Data analyzed (identify specific outcomes) (yes/no). For a paper to be included in the review all of the criteria had to be met. Table 1 displays the definitions used for the inclusion criteria. Once the articles gleaned from the literature search were reviewed, the reasons for exclusion were determined by all of the study authors and recorded for depiction in a flowchart (see Fig 1) for discussion in the results section.

**Table 1:** Inclusion criteria definitions.

Concept	Definition
**Interprofessional Collaborative *Care* (IPC)**	Occurs when healthcare is delivered by intentionally created, work groups that have a collective identity and shared responsibility for a patient or group of patients (e.g., rapid response team, palliative care team, primary care team, and operating room team).
**Interprofessional Collaborative *Practice (IPCP)***	Occurs when multiple healthcare workers from different professional backgrounds work together with patients, families, caregivers, and communities to deliver the highest quality of care. When healthcare providers work collaboratively, they seek common goals and are able to analyze and address any problems that arise. Care is coordinated according to patients’ needs.
**Practice Settings**	***hospital care*:** provide services to diagnose (laboratory, diagnostic imaging) and treat (surgery, medications, therapy) diseases for a short period of time; in addition, they usually provide emergency and obstetrical care ***specialty care*:** provide care for very specific types of diseases; for example, a psychiatric hospital ***nursing homes or long-term care facilities*:** provide long-term care for patients who need extra time to recover from an illness or injury before returning home, or for persons who can no longer care for themselves ***primary Care*:** provide services that do not require overnight hospitalization; the services range from simple surgeries to diagnostic testing or therapy ***home health care*:** provides nursing, therapy, personal care or housekeeping services in the patient's own home ***rehabilitation center*:** provides intensive physical and occupational therapy; includes inpatient and outpatient treatment. ***hospice*:** provides supportive treatment to terminally ill patients and their families
**Peer-Reviewed**	a process by which research for publication is evaluated by a group of experts in the appropriate field
**Data analyzed**	empirical referent that is analyzed in order to draw conclusions about the occurrence of a specific phenomena
**Sample Size**	number of entities (subjects, etc.) in a subset of a population selected for analysis
**Care or practice outcomes defined**	something that happens as a result of an activity or process, e.g., reduced blood pressure, reduced A1c, increased physical activity, reduced length of hospital stay, patient satisfaction, provider evaluation of provided care delivery

**Fig 2:**
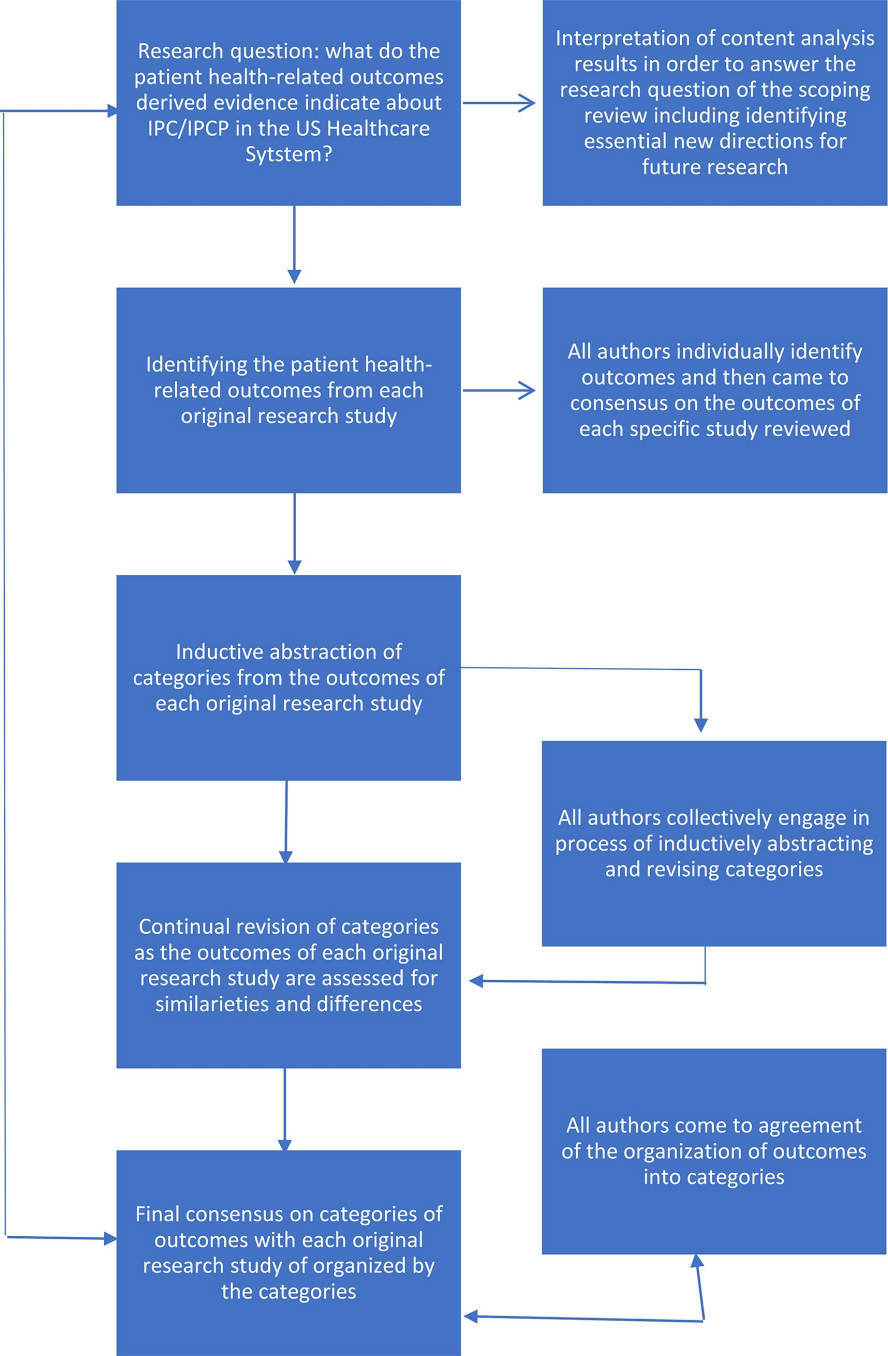
Content analysis process.

***Step 4*:** The evidence presented in the articles reviewed was organized by study design (observational, randomized controlled trial, etc.), the type(s) of data analyzed, the study outcomes, and study results or findings. Organizing the information in this manner allowed for a comprehensive assessment of the reviewed articles. This information is displayed in Table 2 and is discussed in the results section. From this information (now data) categories were derived from the patient outcomes and findings of the included studies. Using a qualitative content analysis approach, study findings were also analyzed and organized into outcome categories by the group consensus of the authors. [18] The process/methodology used to derive categories is depicted in Fig 2 and the categories discussed in the results section.

**Table 2:** Articles meeting inclusion criteria by measured patient outcomes and findings (n = 20).

Included Article	Patient Outcomes Measured*	Conclusions
Anderegg MD, Gums TH, Uribe L, Coffey CS, James PA, Carter BL. Physician-Pharmacist Collaborative Management: Narrowing the Socioeconomic Blood Pressure Gap. Hypertension. 2016;68(5):1314–1320. [19] *Study Design*: clinical trial *Study Setting*: primary care *Study Sample*: 539 patients: 345 received the intervention, and 194 were in the control group *Data Analyzed*: patient records *Professions Engaged*: physicians, pharmacists	to evaluate whether a pharmacist intervention could: - improve blood pressure in high-risk racial minorities and low socioeconomic subjects compared with the control group	- study demonstrated that a pharmacist intervention reduced blood pressure in racial minorities with socioeconomic disparities - blood pressure was reduced by the intervention, however, there were still nonsignificant gaps in mean systolic blood pressure between groups
Arana M, Harper L, Qin H, Mabrey J. Reducing Length of Stay, Direct Cost, and Readmissions in Total Joint Arthroplasty Patients With an Outcomes Manager-Led Interprofessional Team. Orthop Nurs. 2017;36(4):279–284. [20] *Study Design*: Quality Improvement Pre/Post study *Study Setting*: Hospital Care *Study Sample*: 240 THA and 363 TKA patients *Data Analyzed*: patient records *Professions Engaged*: physicians, nurses, pharmacists, dieticians, chaplains, physical therapists, occupational therapists, social workers, care coordinators, home health coordinators,	in the total joint arthroplasty patient population assessed: - direct cost - length of stay - readmissions	- length of stay (total hip arthroplasty [THA] reduced by 0.4 days and total knee arthroplasty [TKA] reduced by 0.6 days) reduced - direct costs (THA reduced by $1,020 per case and TKA reduced by $539 per case) were significantly decreased - 30-day readmission rates of both populations were not significantly increased
Arling PA, Abrahamson K, Miech EJ, Inui TS, Arling G. Communication and effectiveness in a US nursing home quality-improvement collaborative. Nurs Health Sci. 2014;16(3):291–297. [21] *Study Design*: Quality Improvement Pre/Post study *Study Setting*: nursing home care *Study Sample*: not clearly identified *Data Analyzed*: patient records for falls data *Professions Engaged*: not clearly identified	impact of group communication patterns on nursing home residents: - percentage change in the incidence of resident falls	- the rate of new falls declined on average 31% across the facilities in the project
Bingham JT, Mallette JJ. Federal Bureau of Prisons clinical pharmacy program improves patient A1C. J Am Pharm Assoc (2003). 2016;56(2):173–177. [22] *Study Design*: Pre/post intervention study *Study Setting*: prison clinic *Study Sample*: 126 Bureau of Prisons patients with diabetes *Data Analyzed*: patient records *Professions* Engaged: physicians, pharmacists	- A1c in patients with diabetes	- pre intervention measured an average baseline A1c of 10.6% and intervention produced an average decrease in A1c of 2.3%
Dixon DL, Sisson EM, Parod ED, Van Tassell BW, Nadpara PA, Carl D, W Dow A. Pharmacist-physician collaborative care model and time to goal blood pressure in the uninsured population. J Clin Hypertens (Greenwich). 2018;20(1):88–95. [23] *Study Design*: retrospective cohort study *Study Setting*: primary care *Study Sample*: 377 patients (259 = PPCPM; 118 = usual care) *Data Analyzed*: patient records *Professions Engaged*: physicians, pharmacists	- time from the initial visit to the first follow-up visit with a BP <140/90 mm Hg	- median time to BP goal was 36 days vs 259 days in the intervention and usual care cohorts, respectively (P < .001). - At 12 months, BP control was 81% and 44% in the intervention and usual care cohorts, respectively (P < .001)
Gums TH, Carter BL, Milavetz G, Buys L, Rosenkrans K, Uribe L, Coffey C, MacLaughlin EJ, Young RB, Ables AZ, Patel-Shori N, Wisniewski A. Physician-pharmacist collaborative management of asthma in primary care. Pharmacotherapy. 2014;34(10):1033–42. [24] *Study Design*: Prospective pre–post study *Study Setting*: primary care *Study Sample*: 126 patients *Data Analyzed*: patient records *Professions Engaged*: physicians, pharmacists	- the sum of asthma-related emergency department (ED) visits and hospitalizations at 9 months before, 9 months during, and 9 months after the intervention	- Of 126 patients with asthma, the number of emergency department (ED) visits and/or hospitalizations decreased 30% during the intervention (p = 0.052) and then returned to pre-enrollment levels after the intervention was discontinued (p = 0.83) - The intervention reduced asthma-related ED visits and hospitalizations, however, the primary outcome was not statistically significant.
Hackerson ML, Luder HR, Beck AF, Wedig JM, Heaton PC, Frede SM. Addressing primary nonadherence: A collaboration between a community pharmacy and a large pediatric clinic. J Am Pharm Assoc (2003). 2018 Jul—Aug;58(4S):S101-S108.e1. [25] *Study Design*: pre/post intervention study *Study Setting*: primary care *Study Sample*: 59 patients *Data Analyzed*: patient records *Professions Engaged*: pharmacists, primary care prescribers	- nonadherence to filling new prescriptions rates	- increased communication between the primary care provider and the community pharmacy, coupled with targeted patient-specific interventions before the initial fill of medications, resulted in significant reductions in nonadherence
Johnson SW, Ammirati SR, Hartis CE, Weber SF, Morgan MR, Darnell TA, Silwal A, Schmidlin HN, Priest DH. Effectiveness of ledipasvir/sofosbuvir in real-world patients with chronic hepatitis C: a collaborative treatment approach. Int J Antimicrob Agents. 2017 Jun;49(6):778–781. [26] *Study Design*: prospective observational cohort study *Study Setting*: infectious disease care clinic *Study Sample*: 84 patients with chronic hepatitis C *Data Analyzed*: patient records *Professions Engaged*: physicians, pharmacists, nurses	- effectiveness of ledipasvir/sofosbuvir (LDV/SOF) in routine use in clinical practice for the management of chronic hepatitis C virus (HCV)	- of the patients with hepatitis C treated, 97.5% and 91.7% of achieved a sustained virological response (SVR) in the per-protocol analysis and the intention-to-treat analysis, respectively - 2 patients were not cured after relapse of HCV - no patients required LDV/SOF discontinuation - all patients completed the appropriate treatment duration
Kaufman LB, Henshaw MM, Brown BP, Calabrese JM. Oral Health and Interprofessional Collaborative Practice: Examples of the Team Approach to Geriatric Care. Dent Clin North Am. 2016 Oct;60(4):879–90. [27] *Study Design*: 2 patient case studies *Study Setting*: home healthcare *Study Sample*: 2 patients *Data Analyzed*: observations of care outcomes *Professions Engaged*: physicians, dentists, nurses	Case One: 84-year-old homebound man with diagnosis of failure to thrive and history of chronic kidney disease, hypertension, hyperlipidemia, chronic obstructive pulmonary disease, vision impairment (legal blindness), depression, & recent weight loss Outcome: to improve patient’s overall health status Case Two: 80 year old woman with a diagnosis of oral cavity squamous cell carcinoma and oropharyngeal squamous cell carcinoma requiring pre-operative preparation and post-operative dental plan.	Case One: Patient’s health status improved with dental intervention including replaced dentures Case Two: complications with post-operative follow-up revealed problems with not including all relevant interprofessional team members and the importance of ongoing communication between team members in order to ensure that a patient is not lost to follow-up
Ledford JL, Hess R, Johnson FP. Impact of clinical pharmacist collaboration in patients beginning insulin pump therapy: a retrospective and cross-sectional analysis. J Drug Assess. 2013 Jun 19;2(1):81–6. [28] *Study Design*: retrospective and cross-sectional study *Study Setting*: primary care *Study Sample*: 25 patients with diabetes *Data Analyzed*: patient records *Professions Engaged*: physicians, pharmacists	- A1c - Body mass index - number of diabetes-related clinic visits - non-insulin diabetes medication use	- A1c decreased from 8.69 to 7.52% pre and post - BMI decreased from 33.0 to 32.3 kg/m2 pre- and post-intervention - fewer diabetes-related PCP visits were completed post intervention (5.09 vs 3.78 visits/year) - fewer non-insulin diabetes medications were prescribed post intervention
Madan A, Borckardt JJ, Barth KS, Romagnuolo J, Morgan KA, Adams DB. Interprofessional collaborative care reduces excess service utilization among individuals with chronic pancreatitis. J Healthc Qual. 2013;35:41–6. [29] *Study Design*: pre/post intervention *Study Setting*: Hospital Care *Study Sample*: 311patients admitted for treatment of chronic pancreatitis *Data Analyzed*: patient records *Professions Engaged*: medicine, psychology	- LOS - readmission rates - cost of care by healthcare team	- analysis revealed a linear downward trend in LOS (ρ = -0.857, p = .0170) - the interprofessional treatment approach was associated with estimated opportunity cost savings of $670,750.27. - there were no associated changes in 7-, 14-, and 30-day readmission rates, p > .05.
Matzke GR, Moczygemba LR, Williams KJ, Czar MJ, Lee WT. Impact of a pharmacist-physician collaborative care model on patient outcomes and health services utilization. Am J Health Syst Pharm. 2018 Jul 15;75(14):1039–1047. [30] *Study Design*: Pre/post study *Study Setting*: primary care *Study Sample*: 2,480 *Data Analyzed*: patient records *Professions Engaged*: physicians, pharmacists	absolute change in values associated with: diabetes mellitus, hypertension, and hyperlipidemia management from baseline	- Significant improvements (p < 0.01) in glycosylated hemoglobin, blood pressure, low-density-lipoprotein cholesterol, and total cholesterol were observed in the collaborative care group compared with the usual care group. - Hospitalizations declined significantly in the collaborative care group (23.4%), yielding an estimated cost savings of $2,619 per patient.—The return on investment (net savings divided by program cost) was 504%.
Meyers DJ, Chien AT, Nguyen KH, Li Z, Singer SJ, Rosenthal MB. Association of Team-Based Primary Care With Health Care Utilization and Costs Among Chronically Ill Patients. JAMA Intern Med. 2019;179:54–61. [31] *Study Design*: Case/control study *Study Setting*: primary care *Study Sample*: 322 408 patients with chronic conditions *Data Analyzed*: multiple linked administrative databases *Professions Engaged*: not specified	- Outpatient visits - hospitalizations - emergency department - visits, ambulatory care - sensitive hospitalizations - ambulatory care–sensitive—emergency department visits - total costs of care	- Patients in intervention practices experienced a 7.4%increase in annual outpatient visits relative to baseline - after adjusting for patient characteristics there was a statistically significant reductions in hospitalizations, emergency department visits, and in ambulatory care–sensitive emergency department visits - Among patients with less than 2 comorbidities, there was an increase in outpatient visits, hospitalizations, ambulatory care–sensitive hospitalizations - team-based care was not associated with differences in the full patient sample - there were substantial reductions in utilization among a subset of chronically ill patients. - team based care practice transformation in primary care settings may be a valuable tool in improving the care of sicker patients; however, it may lead to greater utilization among healthier patients.
Mior S, Gamble B, Barnsley J, Côté P, Côté E. Changes in primary care physician's management of low back pain in a model of interprofessional collaborative care: an uncontrolled before-after study. Chiropr Man Therap. 2013;21:6. [32] *Study Design*: Pre/post study *Study Setting*: primary care *Study Sample*: 51 patients with lower back pain *Data Analyzed*: patient records *Professions Engaged*: chiropractor, physicians	- number of provider visits - number of prescriptions - number of narcotic prescriptions	- median number of physician visits (2.5 and 1.0), average prescriptions per patients (1.24 and 0.47), total number of narcotic prescriptions (14 and 6) differed between pre-study and study groups - Separate analysis of only the records of low back pain study patients revealed that 61% were referred for chiropractic care during the study period. - Referred patients in the study group had about 25% fewer physician visits and imaging requests.
Nagelkerk J, Thompson ME, Bouthillier M, Tompkins A, Baer LJ, Trytko J, Booth A, Stevens A, Groeneveld K. Improving outcomes in adults with diabetes through an interprofessional collaborative practice program. J Interprof Care. 2018;32(1):4–13. [33] *Study Design*: sequential mixed methods *Study Setting*: primary care (Federally Qualified Health Center) *Study Sample*: 250 patients with diabetes *Data Analyzed*: patient records *Professions Engaged*: medicine, pharmacy, physician assistants	Patient clinical indicators included A1c, glucose, lipid panel laboratory assessments, body mass index, blood pressure, and documentation of annual dental, foot, and eye examinations	- patients who had an A1c of ≥ 7% significantly decreased their A1c (p < .05) and glucose (p < .01) - BMI and annual dental and eye examinations did not improve
O’Leary KJ, Killarney A, Hansen LO, Jones S, Malladi M, Marks K, Shah HM. Effect of patient-centred bedside rounds on hospitalised patients’ decision control, activation and satisfaction with care. BMJ quality & safety. 2016;25(12):921–8. [34] *Study Design*: Cluster randomized controlled trial *Study Setting*: hospital care *Study Sample*: 236 (122 control and 114 intervention unit) patients *Data Analyzed*: research administered surveys *Professions Engaged*: medicine, nursing	- assessed preferred and experienced roles in medical decision-making - compared post-discharge patient satisfaction survey items related to teamwork	- no significant differences in patients’ perceptions of shared decision-making, activation or satisfaction with care were found - analysis found no difference in post-discharge patient satisfaction
Parker RA, Hook LD, Jones ME. Glycemic control: Can nurse practitioners on interprofessional collaborative practice teams enhance clinical outcomes? J Am Assoc Nurse Pract. 2016;28(12):652–658. [35] *Study Design*: cross-sectional study *Study Setting*: primary care *Study Sample*: 120 patients (convenience sample) with diabetes *Data Analyzed*: patient records *Professions Engaged*: nursing, medicine	- A1c	- Patients with two or more FNP visits and two or more visits with the interprofessional care team had statistically significant reductions in A1c levels at the end of 1 year.
Shrader S, Jernigan S, Nazir N, Zaudke J. Determining the impact of an interprofessional learning in practice model on learners and patients. J Interprof Care. 2018 Sep 13:1–8. [36] *Study Design*: Pre/post intervention study *Study Setting*: primary care *Study Sample*: 401 patients with diabetes or depression or hypertension *Data Analyzed*: patient records *Professions Engaged*: clinical psychology, dietetics, medicine, nursing, occupational therapy, pharmacy, physical therapy, social work	- A1c -depression screening scores - blood pressure	- statistically significant results demonstrated A1c values for patients with diabetes were reduced by 0.5% - depression screening improved from 9% to 91% - patients’ hypertension control was similar to baseline
Sisson EM, Dixon DL, Kildow DC, Van Tassell BW, Carl DE, Varghese D, Electricwala B, Carroll NV. Effectiveness of a Pharmacist-Physician Team-Based Collaboration to Improve Long-Term Blood Pressure Control at an Inner-City Safety-Net Clinic. Pharmacotherapy. 2016 Mar;36(3):342–7. [37] *Study Design*: retrospective cohort study *Study Setting*: primary care *Study Sample*: 385 patients with hypertension *Data Analyzed*: patient records *Professions Engaged*: pharmacy, medicine	- Blood pressure	- BP control rate improved to 66% during the first year and persisted throughout the study period, with 68% of patients at goal at the end of the study (p<0.05 compared with baseline)
Sweiss K, Wirth SM, Sharp L, Park I, Sweiss H, Rondelli D, Patel PR. Collaborative Physician-Pharmacist-Managed Multiple Myeloma Clinic Improves Guideline Adherence and Prevents Treatment Delays. J Oncol Pract. 2018 Nov;14(11):e674-e682. [38] *Study Design*: pre/post intervention *Study Setting*: myeloma clinic *Study Sample*: not clearly stated *Data Analyzed*: patient records *Professions Engaged*: Pharmacy, Medicine	1) Improve adherence to treatment and supportive care guidelines 2) reduce delays in receiving oral antimyeloma therapy	- collaborative clinic led to significant improvements in adherence to supportive medications - median time to initiation of bisphosphonate and Pjirovecii pneumonia prophylaxis after autologous transplantation was shortened - the number and duration of delays in obtaining immunomodulatory drug therapy were also significantly reduced.

* Patient outcomes: A1c = Hemoglobin A1c, BP = Blood pressure, LOS = length of stay

***Step 5*:** interpreting the findings. In the *Discussion* section of this paper, the findings of this review were interpreted in light of the contemporary manifestation of interprofessional collaborative practice and/or care in the US.

## Results

Fig 1 illustrates the article selection process for this review. The search conducted in PubMed and Google Scholar yielded 375 papers—249 of which focused on interprofessional collaborative practice and 126 focused on interprofessional collaborative care. After an initial review by one of the study’s authors (MNL), 129 articles tentatively met the study criteria and were then reviewed by the remaining authors. After full examination, a total of 355 articles failed to meet the inclusion criteria. Forty papers were excluded because they were review pieces, 113 papers were not US based, 92 papers did not have patient health-related outcomes, 21 papers did not address interprofessional collaborative practice or interprofessional collaborative care, 54 papers were opinion/commentary or program description papers, 31 papers were duplications, one paper was not peer-reviewed, and three papers were not available from any source. Ultimately, 20 papers [19–38] met the inclusion criteria for review.

Table 2 displays the included papers summarized by study design, study setting, data analyzed and sample size, outcomes studied, and study findings. The selected studies utilized a variety of study designs including clinical trials, quality improvement and pre/post intervention studies. Ten studies were pre/post intervention studies, some of which were quality improvement studies. [20–25, 29, 30, 32, 35, 38] Two studies were clinical trials, [19, 34] one of which was a randomized control trial. [34] Two studies were retrospective cohort studies [23, 37] and one a prospective cohort study. [25] Two studies were cross-sectional, [28, 35] one sequential mixed methods, [33] one a case study,[27] and one a case/control study. [31] In addition to the wide variety of study designs employed, sample sizes ranged widely from one paper reporting two case studies to a sample size [27] of 322,408 patients. [31] The case study project was difficult to assess since each case (two were included in the paper) entailed both a patient along with unspecified multiple healthcare providers as well as family members. [27]

The specific outcomes measured in the studies reviewed varied greatly although some outcomes overlapped among studies. In aggregate, there were 22 distinct outcomes. Among the overlap outcomes displayed in Table 2 are: hospital length of stay, [20, 29] hospital readmission rates, [20, 29] direct cost of care, [20, 21, 31] A1c, [22, 29, 30, 33, 35, 36] blood pressure, [19, 23, 30, 33, 36, 37] and number of office visits. [28, 31, 32] While none of the studies reviewed detected any negative impacts of interprofessional collaborative care or interprofessional collaborative practice, not all findings favored a positive impact of interprofessional care or interprofessional practice.

While study analyses included quantitative, qualitative and mixed methods, descriptive statistical analysis was the most frequently used analytical technique. In all the reviewed studies, at least two professions were engaged with the most frequently engaged professions being physicians (medicine) included in 18 of the 20 studies and pharmacy in 15 of the 20 reviewed studies. Nursing was included in 6 of the reviewed studies. Other engaged professions (e.g., social work, occupational therapy, physical therapy) were represented in one or at most two studies.

The abstracted studies reported on research in six distinct settings: primary care, [19, 23–25, 28, 30–33, 35–37] hospital care, [19, 29, 34] specialty clinic care, [26, 38] nursing home care, [21] home health care, [27] and prison clinic (in and out patient care). [22] The most frequent study setting was primary care followed by hospital care as a distant second.

Finally, an examination of the reviewed studies’ outcomes and findings revealed eight distinct outcome categories. Fig 2 depicts the analysis process and Tables 3 and 4 describe the research outcomes and key study characteristics. The eight categories identified were: 1) Impact of interprofessional collaborative practice on chronic diseases with well-defined management measures [19, 22–24, 28–30, 33–35]; 2) Impact of interprofessional collaborative practice on specialty care outcomes with well-defined management measures [20, 26, 38]; 3) Impact of interprofessional collaborative practice on direct cost of care [20, 29, 31]; 4) Impact of interprofessional collaborative practice on prescribing practices and/or patient adherence [25, 32]; 5) Impact of interprofessional collaborative practice on dental care [27]; 6) interprofessional collaborative practice impact on falls [21]; 7) impact of interprofessional collaborative practice on health services utilization [31, 32]; 8) impact of interprofessional collaborative practice on patient satisfaction. [34]

**Table 3:** Analysis of studies findings from measured patient outcomes.

IPCP impact on patient outcomes (derived from an assessment of the study findings listed in Table 2)	Findings (aggregated from multiple studies)
1) Impact of interprofessional collaborative practice on chronic diseases with well-defined management measures [19, 22–24, 28–30, 33–36]	- pharmacist intervention reduced blood pressure in racial minorities with socioeconomic disparities - intervention produced an average decrease in A1c of 2.3% - emergency department (ED) visits and/or hospitalizations decreased 30% during the intervention (p = 0.052) and then returned to pre-enrollment levels after the intervention was discontinued (p = 0.83) - BMI decreased from 33.0 to 32.3 kg/m2 - fewer diabetes-related PCP visits post intervention (5.09 vs 3.78 visits/year) - fewer non-insulin diabetes medications prescribed post intervention
2) Impact of interprofessional collaborative practice on specialty care outcomes with well-defined management measures [20, 26, 38]	- 97.5% and 91.7% of Hepatitis C patients achieved a sustained virological response (SVR) in the per-protocol analysis and the intention-to-treat analysis, respectively - length of stay (total hip arthroplasty [THA] reduced by 0.4 days and total knee arthroplasty [TKA] reduced by 0.6 days) reduced
3) Impact of interprofessional collaborative practice on direct cost of care [20, 29, 31]	- direct costs (THA reduced by $1,020 per case and TKA reduced by $539 per case) significantly decreased - interprofessional treatment approach associated with estimated opportunity cost savings of $670,750.27.
4) Impact of interprofessional collaborative practice on prescribing practices and/or patient adherence [25, 32]	- increased communication between the primary care provider and the community pharmacy, coupled with targeted patient-specific interventions - significant reductions in nonadherence
5) Impact of interprofessional collaborative practice on dental care [27]	- Collaborative care intervention with dental medicine improved patients health status including replaced dentures
6) interprofessional collaborative practice impact on falls [21]	- new falls declined on average 31% across the facilities in the project
7) impact of interprofessional collaborative practice on health services utilization [31, 32]	- patients in intervention practices experienced a 7.4%increase in annual outpatient visits relative to baseline - intervention practices yielded statistically significant reductions in hospitalizations, emergency department visits, and ambulatory care–sensitive emergency department visits
8) impact of interprofessional collaborative practice on patient satisfaction [34]	- no differences in patients’ perceptions of shared decision-making, activation or satisfaction with care were found - also analysis found no difference in post-discharge patient satisfaction

**Table 4:** Summary of the key study characteristics.

Characteristic	Group	Frequency
Professional teams	2–3 disciplines team	16
	>3 disciplines team	2
	Not clearly identified	2
		
Practice settings	Hospital care	4
	Nursing home	1
	Primary care	12
	Specialty care	3
		
Outcomes	Biometric (HgA1c, BP, Hepatitis, etc.)	17
	Health care cost	3

## Discussion

Interprofessional education is firmly entrenched as an essential component to prepare health professions for a complex and evolving healthcare environment. The Health Profession Accreditors Collaborative states that the need for health professions to work together is unprecedented. [39] However, the merit of interprofessional education is inextricably linked to the value of interprofessional collaboration, making evidence related to the value added proposition of collaborative care critically important. Surprisingly, this scoping review yielded only twenty studies that examined clinically relevant outcomes related to interprofessional collaborative care and interprofessional collaborative practice. Of an original yield of 375 studies published between 2010 and 2018, most studies focused on educational endeavors or provider impressions and not on outcomes documenting clinical impact.

In 2008 Berwick, et al. published their seminal triple aim article [5] which argued that healthcare reform should address patient satisfaction, population health improvement, and rising healthcare costs. Both Berwick’s paper [31] and the US Affordable Care Act, [15] cite interprofessional collaborative care and interprofessional collaborative practice as important components of that reform. However, a 2014 interprofessional practice and education review [3] and 2015 IOM report [1] both noted that few studies examined the impact of interprofessional practice and education on triple aim outcomes—most notably patient health outcomes (or population health). Despite limited evidence, policy makers recommend practitioners develop the knowledge, attitudes, skills and behaviors to work collaboratively. [40] What is encouraging is that while the body of evidence is sparse, most studies examining interprofessional care found it positively impacts care.

Of the 20 research studies reviewed, [19–38] all had at least one patient health-related outcome that could be mapped to a triple aim outcome. Several studies examined patient health condition outcomes (e.g., diabetes, asthma, hypertension) but only three explicitly studied whether the intervention reduced healthcare costs [20, 29, 31] and only one examined the effect on patient satisfaction. [34] No study measured outcomes relevant to *all three* of Berwick, et al.’s, triple aims.

The reviewed studies investigated outcomes across multiple settings, healthcare professions and study designs. The majority found interprofessional collaborative care and/or interprofessional collaborative practice improved health related outcomes such as A1c in patients with diabetes, polypharmacy for pain management in patients with lower back pain, and blood pressure outcomes for patients with hypertension. Healthcare cost savings were documented for chronically ill patients such as those with pancreatitis. The studies reviewed provide blueprints for others wishing to examine the impact of interprofessional collaborative practice or interprofessional collaborative care on appropriate health outcomes. Of note, while not all studies documented benefit, no study found a negative outcome related to interprofessional collaborative care and/or interprofessional collaborative practice,

The majority of the studies included in this review researched outcomes in primary care settings [19, 23–25, 28, 30–33, 35–37] and addressed clearly defined chronic diseases with well-defined management measures. [19, 22–24, 28–30, 33–36] In these instances the challenge of defining, operationalizing, and measuring patient health-related outcomes is minimized.

Of the 20 studies, almost all included medicine and 11 included pharmacy. Surprisingly, no study reviewed included public health professionals, social workers, behavioral health providers or physician assistants and few studies engaged nurses. Only one study included dentists even though their accreditation standards advocate the importance of oral health to overall health and training that links oral health to colleagues in other professions. [41] Addressing these identified gaps in future research will strengthen the evidentiary base of interprofessional collaborative practice. Furthermore, clear articulations of the elements of interprofessional collaborative practice approaches, such as coordination, communication, cooperation, shared decision making and practice [42,43] need to be included in future research.

### Limitations

A number of limitations to this review bear noting. First, the study used very specific definitions for our inclusion criteria. Because the field of interprofessional practice and education has yet to standardize the lexicon of its concepts, the definitions used may have been too restrictive and as a result missed some relevant research. For example, the Cochrane review identified studies with positive patient outcomes from integrated behavioral health care team but the terminology “Collaborative Care” used was differed from the IPCP definition used in this review. Therefore, this review paper was not identified in our initial literature search. While this might affect identification of public health and this work IPCP research studies, we do not believe this substantially changes our review.

In addition, while the US healthcare system is unique, by focusing solely on the US healthcare environment, this review could have potentially missed studies from other developed countries that may have documented pertinent data-driven findings. Finally, the review only used the PubMed and Google Scholar search engines. There is always a possibility that an additional search engine might have yielded additional relevant articles.

## Conclusions

The goal of interprofessional practice and education is to foster care collaboration that optimizes patient outcomes. Although advocates promote the benefits of removing silos among health professionals, there is surprisingly little evidence documenting the health-related outcome benefits of interprofessional collaborative practice and/or care. In addition, our review found inconsistency in use of terminology to describe health care team work and this might have resulted in difficulties identifying all relevant literature. [44] Continued effort to develop common and meaningful terminology and research on the measurable impact of interprofessional collaborative practice and/or care on patient health-related outcomes is needed to document its benefits and to explore the models, systems and nature of collaborations that best improve population health, increase patient satisfaction, and reduce cost of care.

Dedication: Dr. Nawal Lutfiyya unexpectedly passed away before completing the final revisions of this paper. Her co-authors would like to acknowledge her leadership in developing this review and to dedicate this paper in her memory.

## Supporting information

S1 File Table 1. (DOCX)

S2 File Electronic search strategy. (DOC)

S3 File Prisma 2009 checklist. (DOCX)

## References

[1] Institute of Medicine (IOM). Measuring the impact of interprofessional education on collaborative practice and patient outcomes. Washington, DC: The National Academies Press, 2015 26803876

[2] Cox M, Cuff P, Brandt B, Reeves S, Zierler B. Measuring the impact of interprofessional education on collaborative practice and patient outcomes, Journal of Interprofessional Care. 2016; 30: 1–3 10.3109/13561820.2015.1111052 26833103

[3] Brandt BF, Lutfiyya MN, King JA, Chioreso C. A scoping review of interprofessional collaborative practice and education using the lens of the Triple Aim. *Journal of Interprofessional Care*. *J Interprof Care*. 2014; 28: 393–399. 10.3109/13561820.2014.906391 24702046 PMC4162503

[4] Lutfiyya MN, Brandt BF, Cerra FB. Reflections from the Intersection of Health Professions Education and Clinical Practice: State of the Science of Interprofessional Education and Collaborative Practice. Academic Medicine. 2016; 91:766–771. 10.1097/ACM.0000000000001139 26959223

[5] Berwick DM, Nolan TW, Whittington J. The triple aim: care, health, and cost. Health affairs. 2008;27:759–69. 10.1377/hlthaff.27.3.759 18474969

[6] Institute of Medicine (IOM). Crossing the Quality Chasm: A New Health System for the 21st Century. Washington, D.C: National Academy Press; 2001. 25057539

[7] World Health Organization Guidelines 2013: Transforming and scaling up health professionals’ education and training. Geneva, WHO, 2013 Retrieved from https://apps.who.int/iris/bitstream/handle/10665/93635/9789241506502_eng.pdf?sequence=1 26042324

[8] Lutfiyya MN, Brandt B, Delaney C, Pechacek J, Cerra F. Setting a research agenda for interprofessional education and collaborative practice in the context of US health system reform. Journal of Interprofessional Care. 2016;30:7–14. 10.3109/13561820.2015.1040875 26230379 PMC4776700

[9] Interprofessional Education Collaborative Expert Panel. Core competencies for interprofessional collaborative practice: Report of an expert panel. Washington, D.C: Interprofessional Education Collaborative, 2011.

[10] Thistlethwaite J, the GRIN Working Group. Introducing the Global Research Interprofessional Network (GRIN). Journal of Interprofessional Care. 2013; 27: 107–109. 10.3109/13561820.2012.718814 22950790

[11] Haddara W, Lingard L. Are we all on the same page? A discourse analysis of interprofessional collaboration. Acad Med. 2013;88:1–7. 10.1097/ACM.0b013e318279bfbd 23969354

[12] Reeves S, Goldman J, Gilbert J, Tepper J, Silver I, Suter E, Zwarenstein M. A scoping review to improve conceptual clarity of interprofessional interventions. J Interprof Care. 2011;25:167–174. 10.3109/13561820.2010.529960 21182439

[13] Arksey H, O'Malley L. Scoping studies: towards a methodological framework. Int J Soc Res Methodol. 2005; 8:19–32.

[14] Moher D, Liberati A, Tetzlaff J, Altman DG, PRISMA Group. Preferred reporting items for systematic reviews and meta-analyses: the PRISMA statement. Int J Surg. 2010;8:336–341. 10.1016/j.ijsu.2010.02.007 20171303

[15] The Patient Protection and Affordable Care Act: Detailed Summary. Available from: https://www.hhs.gov/sites/default/files/ppacacon.pdf

[16] Bramer WM, Giustini D, Kramer BMR, Anderson PF. The comparative recall of google scholar versus PubMed in identical searches for biomedical systematic reviews: a review of searches used in systematic reviews. Syst Rev. 2013;2:115 10.1186/2046-4053-2-115 24360284 PMC3882110

[17] Wakimoto DK. Google Scholar retrieves twice as many relevant citations as PubMed and provides greater full-text access for quick, clinical nephrology searches. Evid Based Libr Inf Pract. 2014;9:1.

[18] Levac D, Colquhoun H, O'Brien KK. Scoping studies: advancing the methodology. Implement Sci. 2010;5:69 10.1186/1748-5908-5-69 20854677 PMC2954944

[19] Anderegg MD, Gums TH, Uribe L, Coffey CS, James PA, Carter BL. Physician-Pharmacist Collaborative Management: Narrowing the Socioeconomic Blood Pressure Gap. Hypertension. 2016;68:1314–1320. 10.1161/HYPERTENSIONAHA.116.08043 27600181 PMC5063695

[20] Arana M, Harper L, Qin H, Mabrey J. Reducing Length of Stay, Direct Cost, and Readmissions in Total Joint Arthroplasty Patients With an Outcomes Manager-Led Interprofessional Team. Orthop Nurs. 2017;36:279–284. 10.1097/NOR.0000000000000366 28737635

[21] Arling PA, Abrahamson K, Miech EJ, Inui TS, Arling G. Communication and effectiveness in a US nursing home quality-improvement collaborative. Nurs Health Sci. 2014;16:291–297. 10.1111/nhs.12098 24256620

[22] Bingham JT, Mallette JJ. Federal Bureau of Prisons clinical pharmacy program improves patient A1C. J Am Pharm Assoc (2003). 2016;56:173–177. 10.1016/j.japh.2016.01.002 27000168

[23] Dixon DL, Sisson EM, Parod ED, Van Tassell BW, Nadpara PA, Carl D, W Dow A. Pharmacist-physician collaborative care model and time to goal blood pressure in the uninsured population. J Clin Hypertens (Greenwich). 2018;20:88–95. 29237095 10.1111/jch.13150 PMC8031164

[24] Gums TH, Carter BL, Milavetz G, Buys L, Rosenkrans K, Uribe L, Coffey C, MacLaughlin EJ, Young RB, Ables AZ, Patel-Shori N, Wisniewski A. Physician-pharmacist collaborative management of asthma in primary care. Pharmacotherapy. 2014;34:1033–42. 10.1002/phar.1468 25142870 PMC4188693

[25] Hackerson ML, Luder HR, Beck AF, Wedig JM, Heaton PC, Frede SM. Addressing primary nonadherence: A collaboration between a community pharmacy and a large pediatric clinic. J Am Pharm Assoc (2003). 2018;58:S101–S108.e1. 10.1016/j.japh.2018.04.012 29730152

[26] Johnson SW, Ammirati SR, Hartis CE, Weber SF, Morgan MR, Darnell TA, Silwal A, Schmidlin HN, Priest DH. Effectiveness of ledipasvir/sofosbuvir in real-world patients with chronic hepatitis C: a collaborative treatment approach. Int J Antimicrob Agents. 2017;49:778–781. 10.1016/j.ijantimicag.2017.01.016 28389353

[27] Kaufman LB, Henshaw MM, Brown BP, Calabrese JM. Oral Health and Interprofessional Collaborative Practice: Examples of the Team Approach to Geriatric Care. Dent Clin North Am. 2016;60:879–890. 10.1016/j.cden.2016.05.007 27671959

[28] Ledford JL, Hess R, Johnson FP. Impact of clinical pharmacist collaboration in patients beginning insulin pump therapy: a retrospective and cross-sectional analysis. J Drug Assess. 2013;19;2:81–6. 10.3109/21556660.2013.815624 PMC4937654 27536441

[29] Madan A, Borckardt JJ, Barth KS, Romagnuolo J, Morgan KA, Adams DB. Interprofessional collaborative care reduces excess service utilization among individuals with chronic pancreatitis. J Healthc Qual. 2013;35:41–46. 10.1111/jhq.12025 24004038

[30] Matzke GR, Moczygemba LR, Williams KJ, Czar MJ, Lee WT. Impact of a pharmacist-physician collaborative care model on patient outcomes and health services utilization. Am J Health Syst Pharm. 2018;75:1039–1047. 10.2146/ajhp170789 29789318

[31] Meyers DJ, Chien AT, Nguyen KH, Li Z, Singer SJ, Rosenthal MB. Association of Team-Based Primary Care With Health Care Utilization and Costs Among Chronically Ill Patients. JAMA Intern Med. 2019;179:54–61. 10.1001/jamainternmed.2018.5118 30476951 PMC6583420

[32] Mior S, Gamble B, Barnsley J, Côté P, Côté E. Changes in primary care physician's management of low back pain in a model of interprofessional collaborative care: an uncontrolled before-after study. Chiropr Man Therap. 2013;21:6 10.1186/2045-709X-21-6 23369234 PMC3575353

[33] Nagelkerk J, Thompson ME, Bouthillier M, Tompkins A, Baer LJ, Trytko J, Booth A, Stevens A, Groeneveld K. Improving outcomes in adults with diabetes through an interprofessional collaborative practice program. J Interprof Care. 2018;32:4–13. 10.1080/13561820.2017.1372395 29111835

[34] O’Leary KJ, Killarney A, Hansen LO, Jones S, Malladi M, Marks K, Shah HM. Effect of patient-centred bedside rounds on hospitalised patients’ decision control, activation and satisfaction with care. BMJ quality & safety. 2016;25:921–928. 10.1136/bmjqs-2015-004561 26628552

[35] Parker RA, Hook LD, Jones ME. Glycemic control: Can nurse practitioners on interprofessional collaborative practice teams enhance clinical outcomes? J Am Assoc Nurse Pract. 2016;28:652–658. 10.1002/2327-6924.12391 27479873

[36] Shrader S, Jernigan S, Nazir N, Zaudke J. Determining the impact of an interprofessional learning in practice model on learners and patients. J Interprof Care. 2018;13:1–8. 10.1080/13561820.2018.1513465 30212641

[37] Sisson EM, Dixon DL, Kildow DC, Van Tassell BW, Carl DE, Varghese D, Electricwala B, Carroll NV. Effectiveness of a Pharmacist-Physician Team-Based Collaboration to Improve Long-Term Blood Pressure Control at an Inner-City Safety-Net Clinic. Pharmacotherapy. 2016;36:342–7. 10.1002/phar.1710 26917116

[38] Sweiss K, Wirth SM, Sharp L, Park I, Sweiss H, Rondelli D, Patel PR. Collaborative Physician-Pharmacist-Managed Multiple Myeloma Clinic Improves Guideline Adherence and Prevents Treatment Delays. J Oncol Pract. 2018;14:e674–e682. 10.1200/JOP.18.00085 30423263

[39] Health Professions Accreditors Collaborative. Guidance on developing quality interprofessional education for the health professions. Chicago, IL: Health Professions Accreditors Collaborative, 2019.

[40] Bainbridge L, Nasmith L, Orchard C, Wood V. Competencies for interprofessional collaboration. Journal of Physical Therapy Education. 2010; 24:6–11.

[41] Commission on Dental Accreditation. Accreditation Standards for Dental Hygiene Programs. Available from: ada.org/~/media/coda/files/dh.ashx.

[42] Bridges DR, Davidson RA, Odegard RS, Maki IV, Tomkowiak J. Interprofessional collaboration: three best practice models of interprofessional education. Medical Education. 2011; 16: 6035. 10.3402/meo.v16i0.6035 PMC3081249 21519399

[43] Interprofessional Collaborative Practice. Core Competencies for Interprofessional Collaborative Practice: Report of an Expert Panel. Available from: http://www.aacn.nche.edu/education-resources/ipecreport.pdf.

[44] Paradis E, Whitehead CR. Beyond the Lamppost: A Proposal for a Fourth Wave of Education for Collaboration. Acad Med.2018;93: 1457–1463. 10.1097/ACM.0000000000002233 29620672 PMC6159689

