# Supplementary material for: The state of the science of interprofessional collaborative practice: A scoping review of the patient health-related outcomes based literature published between 2010 and 2018
Source: PLoS One. 2019 Jun 26;14(6):e0218578. doi: 10.1371/journal.pone.0218578 (PMC6594675; doi:10.1371/journal.pone.0218578)
Supplement: S1 File — (DOCX) [file pone.0218578.s001.docx]

**S1 Table 1. Inclusion Criteria Definitions**

| **Concept** | **Definition** |
| --- | --- |
| **Interprofessional Collaborative *Care* (IPC)** | Occurs when healthcare is delivered by intentionally created, work groups that have a collective identity and shared responsibility for a patient or group of patients (e.g., rapid response team, palliative care team, primary care team, and operating room team). |
| **Interprofessional Collaborative *Practice (IPCP)*** | Occurs when multiple healthcare workers from different professional backgrounds work together with patients, families, caregivers, and communities to deliver the highest quality of care. When healthcare providers work collaboratively, they seek common goals and are able to analyze and address any problems that arise. Care is coordinated according to patients’ needs. |
| **Practice Settings** | ***hospital care:*** provide services to diagnose (laboratory, diagnostic imaging) and treat (surgery, medications, therapy) diseases for a short period of time; in addition, they usually provide emergency and obstetrical care  ***specialty care:*** provide care for very specific types of diseases; for example, a psychiatric hospital  ***nursing homes or long-term care facilities:*** provide long-term care for patients who need extra time to recover from an illness or injury before returning home, or for persons who can no longer care for themselves  ***primary Care:*** provide services that do not require overnight hospitalization; the services range from simple surgeries to diagnostic testing or therapy  ***home health care:*** provides nursing, therapy, personal care or housekeeping services in the patient's own home  ***rehabilitation center:*** provides intensive physical and occupational therapy; includes inpatient and outpatient treatment.  ***hospice:*** provides supportive treatment to terminally ill patients and their families |

IPC and IPCP definitions derived from: Interprofessional Collaborative Practice. Core Competencies for Interprofessional Collaborative Practice: Report of an Expert Panel. Available from: http://www.aacn.nche.edu/education-resources/ipecreport.pdf.
