# Supplementary material for: The state of the science of interprofessional collaborative practice: A scoping review of the patient health-related outcomes based literature published between 2010 and 2018
Source: PLoS One. 2019 Jun 26;14(6):e0218578. doi: 10.1371/journal.pone.0218578 (PMC6594675; doi:10.1371/journal.pone.0218578)
Supplement: S3 File — (DOCX) [file pone.0218578.s003.docx]

S3: Electronic Search Strategy

We used both MeSH and broader terms in our search to capture all relevant studies. Our search terms used for the review entailed the following:

1) Interprofessional Collaborative Care in the US Healthcare;

2) Interprofessional Collaborative Practice in the US Healthcare;

3) Patient Outcomes and Interprofessional Collaborative Care in the US; and

4) Patient Outcomes and Interprofessional Collaborative Practice in the US.

We used Google Scholar and PubMed search engines for this literature retrieval. We limited our search to the year 2010 to 2018 and literature published in English.
